# Estimation of Drag Finishing Abrasive Effect for Cutting Edge Preparation in Broaching Tool

**DOI:** 10.3390/ma15155135

**Published:** 2022-07-24

**Authors:** Cristian F. Pérez-Salinas, Ander del Olmo, L. Norberto López de Lacalle

**Affiliations:** 1 Faculty of Civil and Mechanical Engineering, Universidad Técnica de Ambato, Ambato 180103, Ecuador; 2 Aeronautics Advanced Manufacturing Centre (CFAA), University of the Basque Country (UPV/EHU), Parque Tecnológico de Bizkaia-Ed.202, 48170 Zamudio, Spain; ander.delolmo@ehu.eus; 3 Department of Mechanical Engineering, ESI Bilbao, University of the Basque Country, 48013 Bilbao, Spain; norberto.lzlacalle@ehu.eus

**Keywords:** cutting edge micro geometry, edge preparation, drag finishing, broaching tool, *R&R* analysis, prediction *ANN*

## Abstract

In recent years, cutting edge preparation became a topic of high interest in the manufacturing industry because of the important role it plays in the performance of the cutting tool. This paper describes the use of the drag finishing *DF* cutting edge preparation process on the cutting tool for the broaching process. The main process parameters were manipulated and analyzed, as well as their influence on the cutting edge rounding, material remove rate *MRR*, and surface quality/roughness (*Ra*, *Rz*). In parallel, a repeatability and reproducibility *R&R* analysis and cutting edge radius *r_e_* prediction were performed using machine learning by an artificial neural network *ANN*. The results achieved indicate that the influencing factors on *r_e_*, *MRR*, and roughness, in order of importance, are drag depth, drag time, mixing percentage, and grain size, respectively. The reproducibility accuracy of *r_e_* is reliable compared to traditional processes, such as brushing and blasting. The prediction accuracy of the *r_e_* of preparation with *ANN* is observed in the low training and prediction errors 1.22% and 0.77%, respectively, evidencing the effectiveness of the algorithm. Finally, it is demonstrated that the *DF* has reliable feasibility in the application of edge preparation on broaching tools under controlled conditions.

## 1. Introduction

Broaching is a machining process used in the manufacture of complex internal and external shapes required by many advanced industries, such as aeronautics, automotive, and marine [1]. The quality of the products manufactured in these industries is of the highest order, where the level of surface and dimensional integrity is commensurate with its critical application. Therefore, the manufacturing suppliers of these industries must comply with such requirements. One of the factors that directly influences the achievement of the desired standards is the cutting tool. Consequently, every process or controllable characteristic that can be applied to a cutting tool has its relevant importance.

The performance of metal removal tools is directly related to the macro and micro geometry of the tool, cutting materials, and coating systems. Macro geometry refers to the geometric shape and angles of the cutting profile. On the other hand, tool micro geometry focuses on the cutting edge. Recent studies show that tool micro geometry has a significant influence on the cutting process [2,3]. In addition, extending the life of the cutting edge is possible if it has a specific shape and quality. Different studies show that such life extension depends on the chip removal operation and cutting conditions [4,5,6]. In this sense, achieving the ideal geometry for each machining process is the main concern of researchers and the manufacturing industry.

There are traditional cutting edge preparation processes that are used by cutting tool manufacturers and other new technologies that are in the development or implementation process [3,7,8,9]. Examples of a traditional processes are *brushing* and *brushing–polishing*, which are widely used by cutting tool manufacturers [10]. A wide range of new tool edge preparation processes are being employed, and each of them has advantages and limitations. One process that gained relevance and great utility in recent years is the planetary drag finishing process [11], it is low cost and has a versatility that allows it to work with the sharpening of complex geometries, which are advantages that give it relevance and utility [12]. However, in the specialized literature, there is little information on the working methodology, its limitations, its accuracy, and its influential parameters in obtaining the cutting micro geometry. In machining, the main purpose of applying the dragging process is to obtain a specific radius on the cutting edge. However, it also brings benefits such as smoothing, polishing, and deburring contours.

The tool cutting edge micro geometry achieved by any edge preparation process has identifiable characteristics, such as the surface topography achieved [4]. Small deformations and chips along the surface of the cutting edge define such topography. The topography at the cutting edge is required to be as sharp as possible, as it affects the surface quality of the machining [3,13]. However, the sharpness depends on the cutting edge preparation process applied. Another important aspect to consider is how fast a given cutting edge radius can be obtained. This is because it is directly related to the costs of the process. For this purpose, the material removal rate (*MRR*) is a metric that helps to visualize the process of obtaining the cutting edge [4,14].

Measurement and test results are always subject to a certain uncertainty. Traditionally, accuracy, linearity, and stability were characteristics considered in the evaluation of measurement systems. However, it is now recognized that important properties, such as repeatability and reproducibility *R&R*, need to be included in the measurement system. *R&R* analysis is applied to several areas of interest, such as the validation of calibration methods or the variability of measurements and instruments, among others. However, it is worth highlighting the fact that it is useful to evaluate the measurement uncertainty and stability of instruments and equipment [15,16]. Consequently, its usefulness is feasible to evaluate the process of obtaining a cutting edge radius by *DF*. To determine whether a measurement system can evaluate the performance of a process, adequate estimates of process variation and measurement variation are needed [16,17].

Due to the rise of Industry 4.0, the inclusion of machine learning for process monitoring is a field of great importance and high demand in different industries. Artificial neural networks are a type of artificial intelligence that allow the prediction of variables. Authors such as [18,19] used the artificial neural network model to study and predict tool wear from machine work parameters. In machining works, such as [18,19,20], *ANNs* were used to predict intentional tool wear (cutting edge preparation). In all these works, small errors in training and prediction were found; therefore, it supports the feasibility of applying this technique to predict the desired radius of the cutting edge. 

This paper describes the use of the drag cutting edge preparation process. The incidence of the process parameters on the response variables (roughness and *MRR*) is studied to obtain valuable information as well as to understand and improve the application of this process. In addition, its accuracy, capability, reproducibility, and limitations are verified. In the end, with the cutting edge rounding results, a *r_e_* prediction was performed by means of an ANN for the purpose of suppressing real tests under experienced system considerations and capabilities. Consequently, the algorithm will help to select rounding conditions for future needs and applications.

## 2. Materials and Methods

In this section, the following aspects are detailed: materials and equipment used, execution of edge preparation tests by dragging, methods for the analysis of experimental results, *R&R* analysis, and prediction of *r_e_*, respectively. 

### 2.1. Materials and Equipment

Abrasive particles play an important role in the removal of material in various stock removal processes. In fact, in abrasive processes, every particle becomes the cutting tool. In addition, the abrasive material, its shape, and size are influential factors in the cutting ability. Therefore, the definition of these parameters is necessary for a good performance in the preparation of cutting edges.

**Figure 1:**
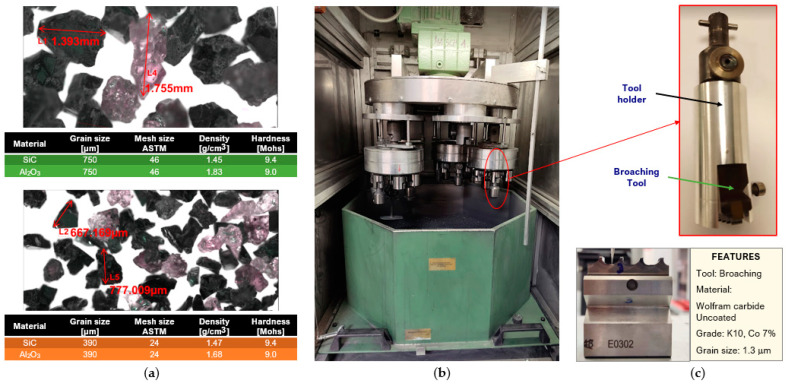
Drag finishing process: (**a**) microscope images of abrasive particles and characteristic, (**b**) drag finishing machine, and (**c**) broaching tool.

Silicon carbide (SiC) and alumina (Al_2_O_3_) abrasive grains were used in two different sizes, 24 and 46, according to the sieve aperture given by ASTM D E 11-70 (Figure 1a).

The cutting tool used for cutting edge preparation (Figure 1c) was an uncoated tungsten carbide broach section for external machining. Several 4-teeth broaching sections were cut by wire electrical discharge machining (*WEDM*) for the different test conditions.

### 2.2. Preparation by Drag Finishing

The cutting edge preparation process was carried out on the German OTEC DF-5 Tools machine (Figure 1b). The methodology for the execution of the experiments consisted of three varying drag depth (*ID*) levels, three abrasive mixing ratios (*RM*) according to the percentage by weight of SiC presence, two grit sizes (*GZ*), and drag duration times (*DT*) to achieve radius ranges recommended in the literature for the broaching process. The radii obtained in broach manufacturing are less than 10 microns. Meanwhile, the rounding of cutting edges depends fundamentally on the material to be machined and the tool material. Studies show that cutting edge radii between 10 and 25 microns improve machining performance on various materials [5]. The different levels for each factor are shown in Table 1. Three measurements were taken for each response variable for each combination.

**Figure 2:**
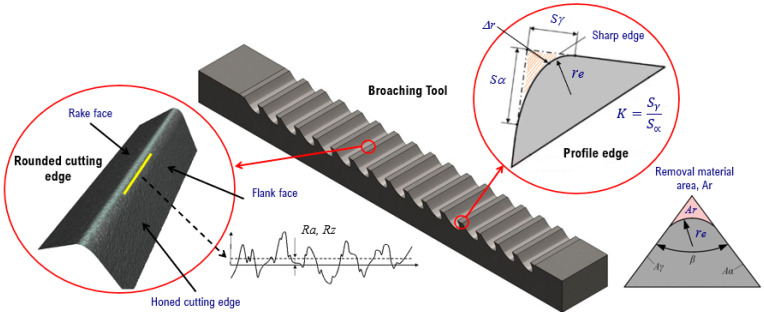
Characterization of the honed cutting edge.

Once the dragging is carried out with the indicated parameters, the prepared cutting edge is characterized (Figure 2). The roundness (*r_e_*) is measured using an Alicona InfiniteFocusG5 profile measuring device. At this point, in addition to extracting the achieved edge radius, the removed area (*Ar*) is also determined geometrically by Equations (1) and (2) [4], for the purpose of observing the behavior with respect to time. Where, *r_1n_* is the nominal initial radius, *r_2n_* is the radius reached, and *K_β_* is the material removal coefficient function of the wedge angle *β* (Figure 2).

(1) $$A_{r}=K_{\beta }\left[r_{2n}^{\;\;\;\;2}-r_{1n}^{\;\;\;\;\;2}\right]$$

(2) $$K_{\beta }=\frac{sin^{2}\left(90{}^{\circ}-\frac{\beta }{2}\right)}{tan\frac{\beta }{2}}-\frac{\pi }{2}\left(\frac{180{}^{\circ}-\beta }{180{}^{\circ}}\right)+sin\left(180{}^{\circ}-\beta \right)$$

Subsequently, the surface roughness (*Ra*, *Rz*) is measured to obtain the surface quality obtained in each condition. For the determination of the roughness profile, a high pass of the main profile was filtered with a cut length of 80 μm. Finally, to contrast the surface topography achieved at the cutting edge, images of the treatment zone were captured. With the same equipment, the profile radius measurement was performed with a 10× lens, and both the topographic inspection and roughness measurement were performed with a 50× lens. 

### 2.3. Repeatability and Reproducibility Analysis, R&R

The guidelines for the calculation of repeatability and reproducibility (*R&R*) are given by ISO 5725-2:2019. *Process variation* is composed of part-to-part and measurement variation. It can be estimated from a large sample of historical data or using the parts included in the study. In the absence of historical data, 24 parts or measurements are used, which is greater than the typical requirement of 10 parts. *Measurement variation* is estimated from the parts, reproducibility, and repeatability. In addition to the 24 parts chosen, measurements with 3 operators are used, a typical requirement of the *R&R* analysis. The 3 technicians performed the measurement of the *r_e_* randomly from the parts. The 24 parts are composed of *r_e_* measurements at DF times of 0, 20, 30, and 40 min at 3 immersion depth and 2 different locations. Three replicates of the 24 measurements are performed. Therefore, each technician performs 72 measurements for a total of 210 measurements.

### 2.4. Cutting Edge Radius Prediction

**Figure 3:**
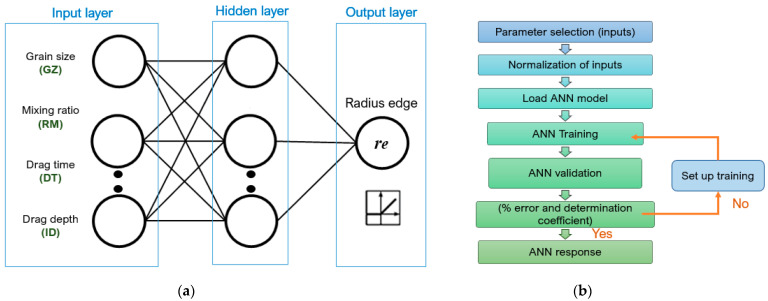
Characterization of the honed cutting edge. (**a**) network layout; (**b**) flowchart.

Based on the experimental results achieved, they are discussed, and a preparation radius prediction is applied by means of artificial intelligence (artificial neural network *ANN*). The *ANN* architecture is shown in Figure 3a; in the input layer there are 4 neurons and in the output layer only one neuron, which represents the cutting edge radius. To facilitate training and balance the importance of each parameter, the database was normalized between values 0 and 1. The experimental data set used for the training stage was divided by cross validation into: 80% for training and 20% for validation. It was developed with a feed-forward and backpropagation neural scheme on the total experimental data. The methodology applied for the estimation of cutting edge radius is described in Figure 3b.

The Equation (3) [21], is used to determine the synaptic connection weights, where *CW′_ij_(n*) is the new connection weight between node “*i*” and node “*j*” of the previous layer and Δ*W_ij_*(*n*) is the synaptic weight correction.

(3) $${{CW}^{\prime }}_{ji}\left(n\right)=W_{ji}\left(n\right)+\Delta W_{ji}\left(n\right)$$

For the evaluation of the evaluation performance, [22] recommends using the mean absolute error *MAE*, the mean squared error *MSE*, and the coefficient of determination *R*^2^. These metrics are presented in Equations (4), (5), and (6), respectively.

(4) $$MAE=\frac{1}{n}\overset{n}{\underset{i=1}{\sum }}\left|\left(y_{exp}-{\hat{y}}_{pred}\right)\right|$$

(5) $$MSE=\frac{1}{n}\overset{n}{\underset{i=1}{\sum }}{\left(y_{exp}-{\hat{y}}_{pred}\right)}^{2}$$

(6) $$R^{2}=1-\left(SSE/SST\right)$$

where, *y_exp_* represents the experimental values, and ${\hat{y}}_{pred}$ expresses the predicted values, *SSE* and *SST* are the sum of squares of the residuals and the total sum of squares, respectively.

## 3. Results and Discussion

This section details the results of the behavior and influence of the factors, the orientation of the tool on *r_e_*, the results of the surface quality, the results of the *R&R* analysis, and the accuracy of the prediction through the artificial neural network.

### 3.1. Preliminary Tests, Influence and Behavior of Factors

**Figure 4:**
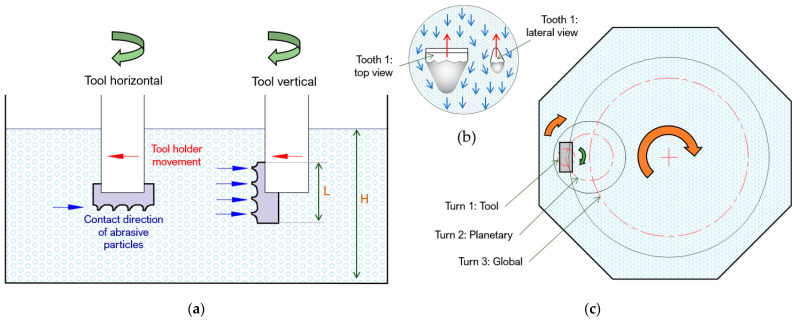
(**a**) The mounting of the broaching tool with different orientations, (**b**) the movement of abrasive particles against the first dragged tooth in horizontal orientation, and (**c**) relative movements of the tool in the drag finishing process.

The rounding results are analyzed in two possible locations for the tool (Figure 4a). The location of the broach will depend on its dimensions, being the most predominant length. In the industry, there is a diversity of lengths for broaches for the purpose of being able to be of great length and to be mounted in sections. Depending on the length *L*, the natural and practical location will be the vertical one, but it will be limited by the capacity of the container of the abrasive grain, especially its height (*H*).

**Figure 5:**
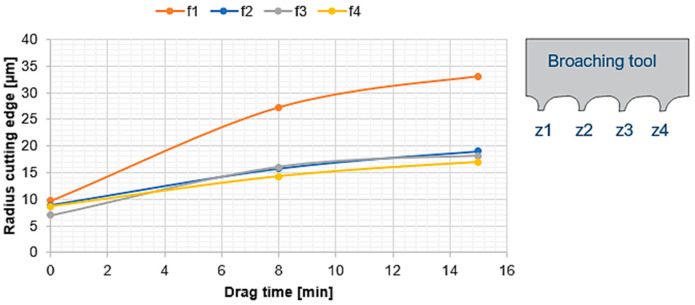
Results of the four cutting edge rounding of the broaching tool, drag finishing in a horizontal position.

However, a test was carried out to see the development of the rounding in a horizontal position. It was verified that the first cutting edge in contact with the abrasive particles is the one that suffers the greatest wear or rounding (Figure 5). This may be due to the fact that the first tooth absorbs the greatest impact energy with the particles as it opens its way in the dragging process (Figure 4b). However, the other cutting edges become uniformly rounded over time.

**Figure 6:**
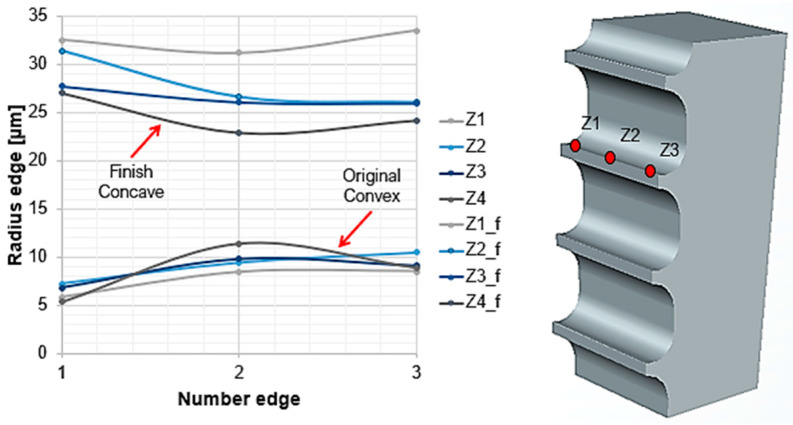
Cutting edge radius measurement at three different points along the cutting edge.

Due to the rotation on its own axis and the planetary-type rotation, along the cutting edge on the broach it changes from a convex to a concave shape (Figure 6). The convex shape is the shape obtained from the original manufacture of the tool, and the concave shape is the result of rounding by drag finishing after 30 min of operation.

**Figure 7:**
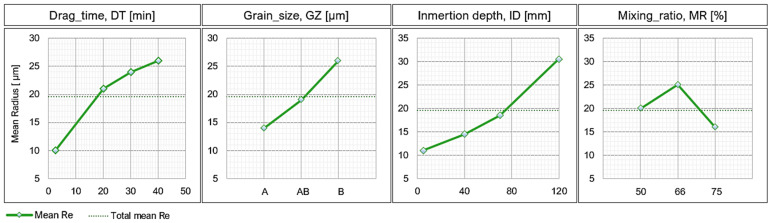
Effect of experimental parameters on cutting edge radius.

The second part of the tests consisted of an in-depth analysis of the rounding evolution of the cutting edge in a vertical orientation, using tool blades at different heights as reference points. Figure 7 shows the incidence of each quantitative parameter (factors) manipulated in the experiment on the cutting edge radius, *r_e_* (response). It can be observed that in no case is the behavior of the response variable linear, as it is increasing both for *DT*, *GZ*, and *ID.* In general, all the factors have a significant influence on *r_e_*. Therefore, the control of each of them is important when obtaining a specific *r_e_* in the tool.

As for the time factor *DT*, the influence increases as time increases, reaching a radius of 26 µm. The influence of grain size (*GZ*) is directly related to *r_e_*, i.e., a large GZ gives a large *r_e_*, while a smaller GZ gives a small *r_e_*. The combination of abrasive grain sizes resulted in obtaining an approximate average *r_e_*. The general trend of *r_e_* as a function of ID is increasing. The trend of the curve indicates that with a deeper depth the radius can grow exponentially. However, the maximum depth of the tank limits the depth of the process and the amount of abrasive material. To minimize *r_e_* variations, the particle mixer should be used whenever possible. This element uniforms the mixture and avoids the segregation of particles of different sizes or the appearance of the nutshell effect [23,24]. Finally, the effect of the SiC percentage on the cutting edge radius of the tool showed a particular behavior. At a SiC percentage in the mixture *RM* of 66%, a larger *r_e_* is obtained than that obtained with an *RM* of 50% and 75%, respectively, being the *RM* of 75% with which relatively smaller radius edges were obtained. 

**Figure 8:**
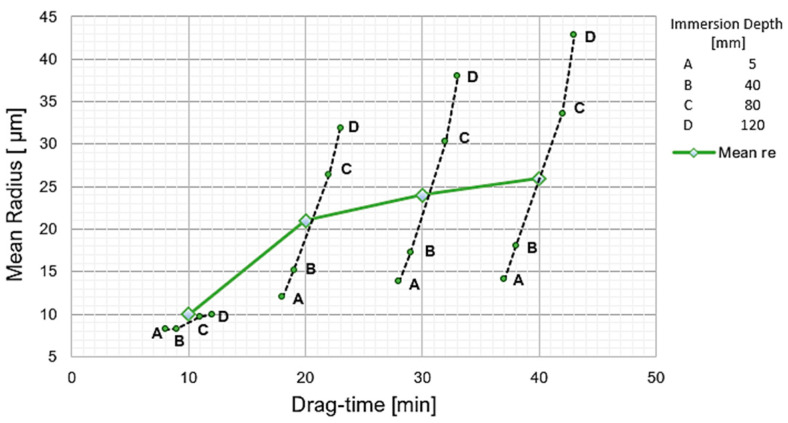
Combined effect of the drag time DT and the drag depth ID on the cutting edge radius *r_e_*.

The combined effect of *DT* and *ID* on *r_e_* can be observed in Figure 8. In the first instance, the average values of the *r_e_* increase over time are shown, starting from an original tool radii between 7 and 9 μm, and reaching a final radius of 26 μm. On the other hand, the progress of *r_e_* obtained at different *ID* depths from 5 to 120 mm is shown, represented by points *A* to *D*, reaching a maximum radius of 43 µm. It follows from the above that the combined effect of *DT* and *ID* on *r_e_* has a direct incremental relationship, but it is not linear.

**Figure 9:**
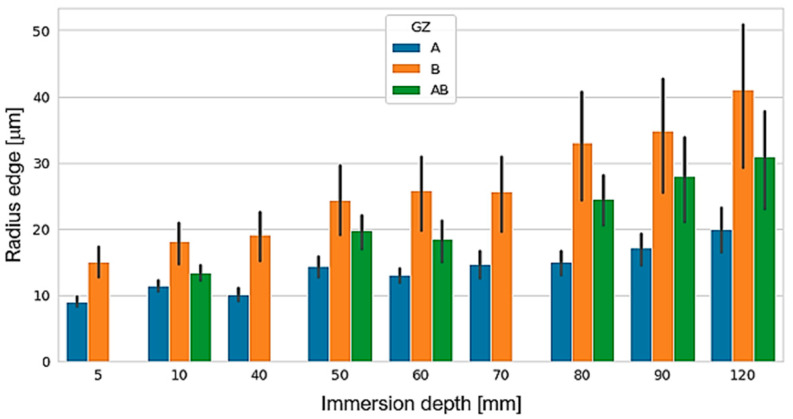
Combined effect of grain size and drag depth on cutting edge radius *r_e_*.

The combined effect of *GZ* grain size and *ID* drag depth causing an increase in *r_e_* is shown in Figure 9. For each *GZ*, the increase in *r_e_* increases in different proportions as we increase *ID*. As an example, for combination *A*, the radius size is doubled from the initial radius to the final radius, and for combination *B*, the radius reached is about three times the initial radius. In industrial application, this result can be translated into different cutting edge preparation control possibilities. With combination *B*, the largest cutting edge radii could be obtained at any depth compared to the other two combinations. On the other hand, if a fine control of the cutting edge radius increment is desired, combinations *A* and *AB* will allow this control. This is possible primarily because of the size of the abrasive grain. That is, the larger the grain size, the greater the cutting edge and cutting capacity [14]. In addition, it is understood from physics that, as the immersion depth increases, the greater the pressure of the grain surface interaction of the cutting edge, which will contribute to greater material removal. 

**Figure 10:**
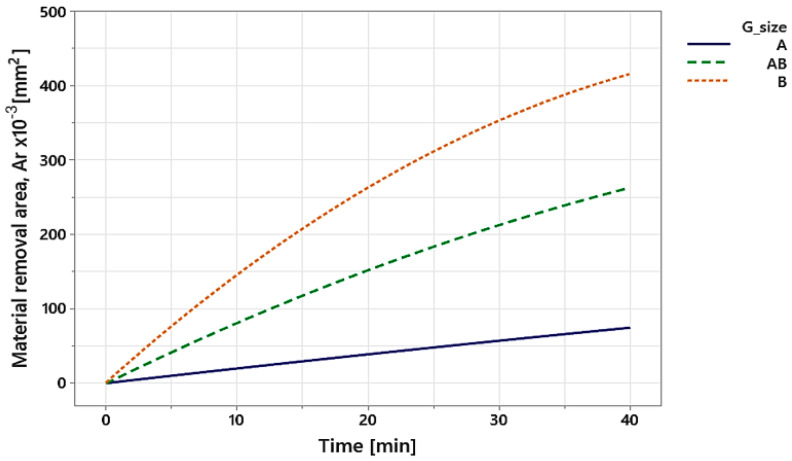
Material removal area of the three abrasive blends with time variation.

The result of the characterization of the removal process for the cutting edge preparation is shown in Figure 10. The trend of *Ar* versus the combined effect of *DT* and *GZ* is the result of the average of all tested combinations (Table 1). As *Ar* is directly dependent on the increase in radius (Equation (1)), the area removed, and consequently the material removal rate, will increase in such a way that the particle removal aggressiveness of the abrasive grains is increased. That is the reason why the abrasive grain mixture *B* is larger than *AB* and *A*, respectively. It also depends directly on the coefficient *K_β_*, but when analyzing the same cutting edge, it becomes a constant value, as it is directly dependent on the wedge width *β*.

### 3.2. Surface Roughness Analysis at the Cut Edge

**Figure 11:**
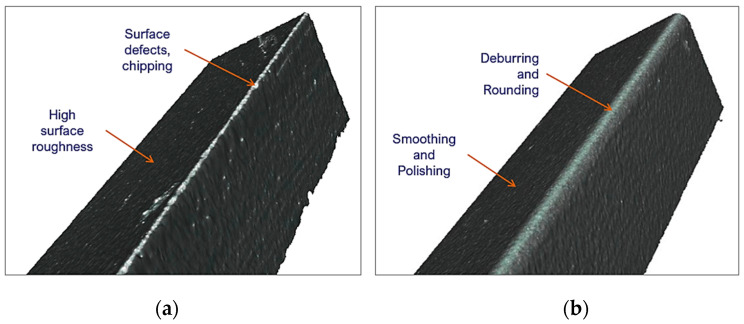
Improvement of surface defects at the cutting edge, (**a**) original factory surface, (**b**) post-treatment improved surface.

Residual surface defects from the manufacturing process at the edges can reduce the quality of a workpiece. By applying the drag finishing process, these imperfections are eliminated and reduced, as in the case of surface roughness (Figure 11). Consequently, it will provide a smooth subsequent finishing and better handling.

**Figure 12:**
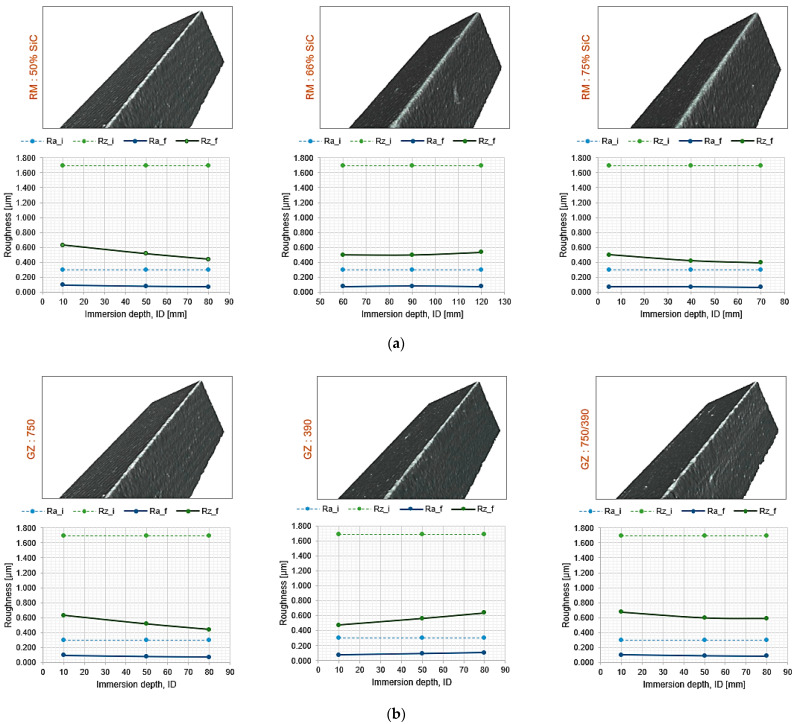
Behavior of the surface roughness against, (**a**) percentage of silicon carbide *RM*, (**b**) abrasive grain size *GZ*.

The roughness achieved on the surface of the *r_e_* cutting edge radius by the drag finishing process versus the immersion depth is presented in Figure 12. Where, the subscripts i and f correspond to the initial and final roughness, respectively. For all the cases shown, the reduction in the surface roughness is remarkable. At the same time, there is greater uniformity in *Ra* and greater variability in *Rz*. By definition, *Rz* is more sensitive to the detection of imperfections in the machined surface; therefore, it is widely used for the control and monitoring of surface irregularities [25,26,27]. However, the *Rz* values obtained by drag finishing are lower or equal to those originally obtained in the manufacture of cutting tools by grinding operation [25,26]. Considering the effect produced by the percentage of SiC in the abrasive mixture (Figure 12a), it can be observed that lower SiC content allows for achieving a higher *Rz* compared to the higher inclusion of SiC in the mixture. This means that the presence of 50% alumina in the mixture has a higher abrasive capacity. Similarly, the effect of abrasive grain size on surface roughness is shown in Figure 12b. It is observed that there is no significant variation in the different abrasive grit sizes used, but there is a slight reduction when using a 750 grit rather than a 390 grit. A particular opposite effect occurs between the two grit sizes used as the immersion depth increases, which should be studied further to know if the behavior is maintained or is a particular case of the tested conditions. However, that proved to be the great advantage of applying drag finishing for edge rounding with a higher surface quality. This is clearly seen in the reduction in surface roughness by three times *Ra* and greater than two times *Rz* for all cases.

To illustrate the resulting defects, Figure 12 shows images of the cut edge, where the dullness measurements were performed. The presence of disturbances and chipping is identified in certain areas of the cutting edge, which implies that these defects are the result of the original treatment by grinding and are replicated by the process applied in this study. Finally, as for *Ra*, it can be observed that its value tends to be constant, regardless of *ID*.

### 3.3. Repeatability and Reproducibility R&R Analysis

The results of the repeatability and reproducibility *R&R* analysis are shown in Table 2. The analysis indicates that the contribution of repeatability is greater than reproducibility in obtaining the *r_e_* of the process.

**Figure 13:**
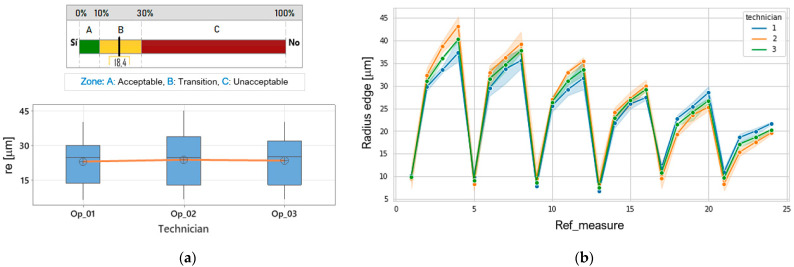
Measurement system capacity, (**a**) determination zones, general rule, (**b**) variability of measurement by technician.

Table 2 shows the sources of variation in the measurement system in obtaining the edge radius *r_e_*, as well as the percentage of contribution to the variance. First, it shows us that the measurement system variation is equal to 18.49% of the process variation. This indicates that the system is in the marginal zone (Figure 13a), where acceptance is possible as long as its limitations, the importance of the application, and the cost are known [19,20].

Second, it was found that the repetition component of each test (repeatability) has a measurement variation of 76.7% and represents 14.18% of the total variation of the process; on the other hand, the component referring to the operators or technicians (reproducibility) has a variation in the measurement of 64.2%, which represents 11.86% of the total variation of the process. In practice, it means that the measurements obtained by the three technicians are not noticeably scattered. This is illustrated in Figure 13b, where it is observed that the measurements of operators 1 and 3 are similar, while operator 2 has the greatest relative variability among technicians.

The result found in the present analysis is consistent with the criteria given by [12], where it is stated that the “Drag Finishing” process is an applicable cutting edge preparation method for improving cutting performance in terms of accuracy and repeatability.

### 3.4. Cutting Edge Radius Prediction by ANN

**Figure 14:**
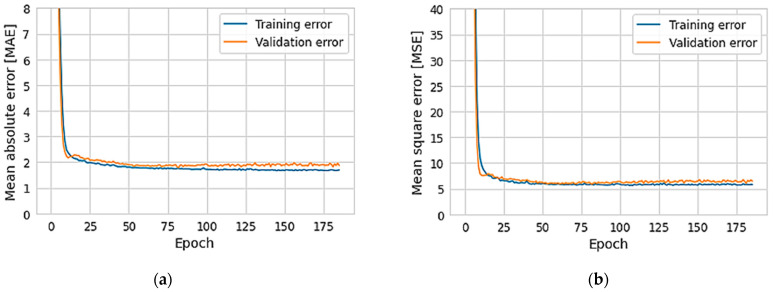
*ANN* application prediction errors, (**a**) mean absolute error *(MAE)*, (**b**) mean squared error *(MSE)*.

This section presents the results of the prediction of *r_e_* by *ANN*, machine learning by the supervised learning method, with the backpropagation gradient descent training algorithm. A maximum number of interactions (epochs) of 1000 was chosen. Cross validation was used to improve the generalization capability. A data set of 324 *r_e_* measurements was obtained as input. Eighty percent of the data were used for training and 20 for validation. It took 141 epochs to find the best training. This means that the errors are no longer reduced, but stabilized (Figure 14). A coefficient of determination of 0.961 with a standard deviation of 0.00631 was obtained. Therefore, the prediction was continued.

The *ANN* prediction results are summarized in Table 3. Depending on the mean absolute error *MAE*, mean square error *MSE*, and the coefficient of determination, an optimal network architecture was arrived at. Figure 14 shows the variation in training and validation errors with the number of iterations for the network used in the present study.

**Figure 15:**
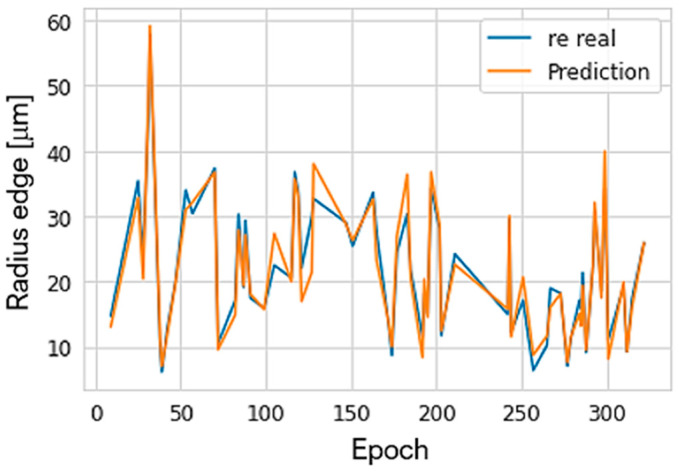
Comparison of the experimental radius with that predicted by *ANN*.

The *r_e_* predicted by the neural network was compared with the corresponding experimental values and is shown in Figure 15. In addition, the average percentage of prediction errors was found to be 9.33% compared to the actual experimental values of the shear edge radius.

## 4. Conclusions

In this paper, the performance of an abrasive mixture of SiC and Al_2_O_2_ at different grain sizes and percentages of mixture are used to prepare uncoated tungsten carbide broaching tools by a drag finishing process. Important parameters, such as time and drag depth were controlled to determine the evolution of cutting edge rounding, material removal rate, repeatability and reproducibility, surface topography, and develop a prediction model by *ANN*. The important conclusions are:

- The parameters incident to obtaining a cutting edge radius were, in order of importance: plunge depth, dragging time, abrasive mix percentage, and abrasive size.
- The incidence of tool location is very important in obtaining a specific radius cutting edge value. In this process, location is understood as the positioning angle and depth of dragging. As for the positioning angle, the horizontal positioning of the broach during dragging causes a superior rounding on the first cutting edge (33 μm) which can be up to two times larger than the radius of the other cutting edges (18 μm). On the other hand, depending on the depth of dragging, the radius of the cutting edge increases in a progressive non-linear way. On average, the growth ranges from 12 μm at 5 mm depth to 31 μm at 120 mm depth. As far as roughness is concerned, it could be identified that the incidence is greater for the abrasive inclusion rate than the grain size. The substantial reduction in surface defects by *Ra* and *Rz* are a third of its original measure. On average from initial Ra: 0.3 microns to final Ra: 0.09 microns, and from initial Rz: 1.7 microns to final Rz: 0.5 microns.
- In terms of accuracy of the reproduction of the tool cutting edge radius, it is very acceptable compared to traditional processes, such as brushing and blasting. Obtaining from the *R&R* study of the reproducibility source a standard deviation of 1.22 that corresponds to 11.86% of the process variation.
- The prediction accuracy of the preparation radius with *ANN* was 93.7%, which demonstrates the effectiveness of the algorithm.
- The limitation of the *drag finishing* process is essentially related to the tool dimensions. In this case, long broaches would make it difficult to locate, hold, and therefore reproduce the geometry of the cutting edge on all the teeth.

## Figures and Tables

**Table 1:** Factors levels for experimental application.

Factors	I	II	III
Abrasive type	SiC	Al_2_O_3_	-
Grain size [µm]	390 (A)	390 + 750 (AB)	750 (B)
SiC mixing ratio [%]	50	66	75
Drag time [min]	10/10	15/15	20/20
Drag depth [mm]	40	60	80

**Table 2:** Characterization of the honed cutting edge.

Fuente	Standard Deviation (SD)	Study Variation (6 × SD)	% Study Variation (% SV)	Variance Component (CV)	% Contribution (CV)
Gage *R&R* total	1.9132	11.4793	18.49	3.660	3.42
Repeatability	1.4676	8.8058	14.18	2.154	2.01
Reproducibility	1.2274	7.3643	11.86	1.506	1.41
Technician	0.2474	1.4842	2.39	0.061	0.06
Technician*Ref_measure	1.2022	7.2132	11.62	1.445	1.35
Part to part	10.1703	61.0216	98.28	103.434	96.58
Total variation	10.3487	62.0920	100.00	107.095	100.00

**Table 3:** Summary of ANN prediction results.

Data Set	*MAE*	*MSE*	*R* ^2^
Training	0.0162	0.0585	
Validation	0.01869	0.0643	0.937

## Data Availability

Not applicable.
